# Potential Release of Zinc and Cadmium From Mine-Affected Soils Under Flooding, a Mesocosm Study

**DOI:** 10.1007/s00244-020-00777-0

**Published:** 2020-11-11

**Authors:** Elio Padoan, Aline Hernandez Kath, Ledemar Carlos Vahl, Franco Ajmone-Marsan

**Affiliations:** 1 Dipartimento di Scienze Agrarie, Forestali e Alimentari, Università Degli Studi Di Torino, Largo Paolo Braccini 2, 10095 Torino, Grugliasco, Italy; 2 Soil and Water Management and Conservation Program, University Federal of Pelotas, Pelotas, Brazil

## Abstract

Metal-contaminated mining soils pose serious environmental and health risks if not properly managed, especially in mountainous areas, which are more susceptible to perturbation. Currently, climate change is leading to more frequent and intense rain events, which cause flooding episodes, thereby altering soil redox equilibria and contaminants stability. We evaluated the potential release of Zn and Cd (two of the most common inorganic contaminants) and the factors regulating their solubility and speciation in two heavily contaminated soils representative of a Zn-mining area. The soils were flooded under aerobic (for 24 h) and anaerobic (for 62 days) conditions using mesocosm experiments, sequential extractions, and geochemical modelling. Leaching trials under aerobic conditions showed a high release of Zn and Cd (10 times the legislative limits), with metals possibly migrating via water infiltration or runoff. Under anaerobic conditions Zn and Cd were initially released. Then, solution concentrations decreased gradually (Zn) or sharply (Cd) until the end of the experiment. Sequential extractions and multisurface modelling indicated that both metals precipitated mainly as carbonates. This was confirmed by a geochemical multisurface modelling, which also predicted the formation of sulphides after 60 days in one soil. The model calculated metals to be preferentially complexed by organic matter and well predicted the observed soil solution concentrations. The results showed that during flooding episodes contaminants could be promptly transferred to other environmental compartments. The use of multisurface modelling coupled with laboratory experiments provided useful indications on the potential release and speciation in case of anoxic conditions.

**Electronic supplementary material:**

The online version of this article (10.1007/s00244-020-00777-0) contains supplementary material, which is available to authorized users.

Mining activities have had a considerable role in the pollution of the environment and are one of the most important point-source of soil and water contamination (García-Carmona et al. 2017; Zhu et al. 2019). In particular, metal mining can cause serious health risks as metals are usually toxic at low concentrations and not degradable, thus remaining in soil and water environments for long periods (Alloway 2013; Langård 2015; Padoan et al. 2019; Rafiee et al. 2020; Mokhtarzadeh et al. 2020).

The potential exploitation of a mine depends on the price of the commodity and on the available technology. In the past, large amounts of mining residues of variable metal concentration have been abandoned in the vicinity of the mining site due to the limitations of the technology and environmental legislation. In recent years, a number of cases have been described and investigated worldwide (Table 1), testifying to the growing concern of the potential risk to human health and to the surrounding ecosystems.

**Table 1:** Selected recent studies on contamination from potentially toxic elements due to mining residues, along with the concentration range of the contaminants

Location	Commodity	Soil contaminants (mg kg^−1^)	Reference
Brazil	Zinc, lead, silver	Pb (2300–5700); Zn (698–2960)	Puga et al. 2015
Chile	Copper, gold	Cu (39–6740); Pb (8–2670; Zn (45–2280)	Reyes et al. 2019
China	Zinc, lead	Cd (9–18); Pb (1305–2995); Zn (2899–4878)	Zhu et al. 2019
Congo	Copper, cobalt	As (5–233); Cd (1–120); Co (2–22,467); Cu (11–126,013); Pb (1–1666); Zn (11–21,886)	Pourret et al. 2016
Germany	Zinc, lead	As (16–267); Pb (294–13,789); Zn (207–7426)	Antoniadis et al. 2017
Italy	Zinc	Cd (13–493); Pb (98–393); Zn (9000–132,000)	Padoan et al., 2020
Kansas (USA)	Zinc, lead	Cd (67); Pb (5048); Zn (23,468)	Karna et al. 2016
Spain	Pyrite	As (127–270); Pb (210–479); Zn (242–277)	García-Carmona et al. 2017
Tunisia	Phosphate	Cd (5–85); Cr (83–474); Zn (66–547)	Khelifi et al. 2019
Uganda	Copper	Co (80–148); Cu (165–10,217)	Abraham and Susan 2017

Italy has seen thriving mining activity in the past, but it is now mostly dismissed and has left several extended contaminated sites (Dino et al. 2018; Mehta et al. 2019), especially in mountain areas, where the environment is more susceptible to perturbation. The impending risk of transfer of PTE to other environmental compartments may be exacerbated by the currently changing climatic conditions. More frequent and intense extreme rain events cause more flooding episodes (EASAC European Academies’ Science Advisory Council 2018; Sánchez-Rodríguez et al. 2019; Blöschl et al. 2019). This would alter the redox equilibria controlling the solubility of PTE in soils and their eventual release to the water system, especially in a situation where they are associated with compounds sensitive to redox fluctuations (Grybos et al 2007; Khaokaew et al. 2011; Ajmone Marsan et al. 2019). Mining areas can become a source of PTE susceptible to be released to the surrounding soils and water if the chemical equilibria of the system are altered. Monitoring of abandoned mining sites is therefore mandatory to prevent or mitigate toxic consequences (García-Carmona et al. 2017).

When a PTE-contaminated soil is flooded, a number of chemical reactions may occur that will define whether these metals will be retained or released. A metal under reducing conditions can be adsorbed/desorbed onto solid components, coprecipitated onto hydrous oxides of Fe and Mn, precipitated as a carbonate and/or as an insoluble sulphide, or complexed by organic matter (Popescu et al. 2013; Balint et al. 2014; Karna et al. 2017).

The risk posed by PTE release from the soil is less related to their intrinsic properties than it is to the possible solubilization of the components holding them into the soil. An abrupt decrease in the redox potential would cause the solubilization of Mn and Fe oxides and the metals that are associated to them, either coprecipitated or adsorbed on their surface, would be released (Violante et al. 2010; Kögel-Knabner et al. 2010; Wang et al. 2019). The organic matter (OM) also influences metals speciation through adsorption, coprecipitation, and complexation. Thus, the reductive dissolution of OM could release the associated trace metals (Ren et al. 2015; Pan et al. 2016). In neutral and acidic soils, the reduction of nitrate, Fe, and Mn leads to an increase in pH, which adds to the organic matter alteration (Zhu et al. 2020).

Studies have reported the effect of redox conditions on metals solubility. Grybos et al. (2007) observed that the fate of various metals was linked to that of Fe and Mn in soil nodules. Similarly, Du Laing et al. (2009) reported that Fe and Mn oxides were the main carriers of Cd and Zn in oxic environments. In fact, iron behaviour in changing redox environments has been used for choosing soil remediation technologies (Cundy et al. 2008).

For Cd, decreases of its bioavailability under anaerobic conditions has been attributed to the formation of CdS (Wang et al. 2019). However, the work from Khaokaew et al. (2011) in paddy soils showed that CdS is not the only salt that controls Cd solubility in flooded soils, because this metal is associated with several others mineral phases, including kaolinite, ferrihydrite, and humic acids. The authors investigated Cd speciation and found that carbonate species, such as Cd–CaCO_3_ and Cd–CO_3_, dominated in the flooded soil. Investigating rice paddy soils contaminated by a nearby mine, Yu et al. (2016) observed that Cd behaviour was related to Fe oxides solubility and soil pH. More recently, Wang et al. (2019) confirmed that the mobilization of Cd in paddy soils depends on either Fe–Mn redox dynamics or on changes in pH of the solution. Similarly, Cui et al. (2020) observed an immobilization of Cd and Cu by hematite under flooding of a red soil.

Our work evaluated the potential release of metals subsequent to a flooding in soils from a former Zn-mining area, heavily contaminated and enriched in Zn and Cd due to the inappropriate management of mining residues. The goals were to elucidate the factors regulating Zn and Cd solubility and speciation in the soil solution, using laboratory experiments, chemical extractions, and multisurface modelling.

## Materials and Methods

### Study Area and Sampling

**Fig. 1:**
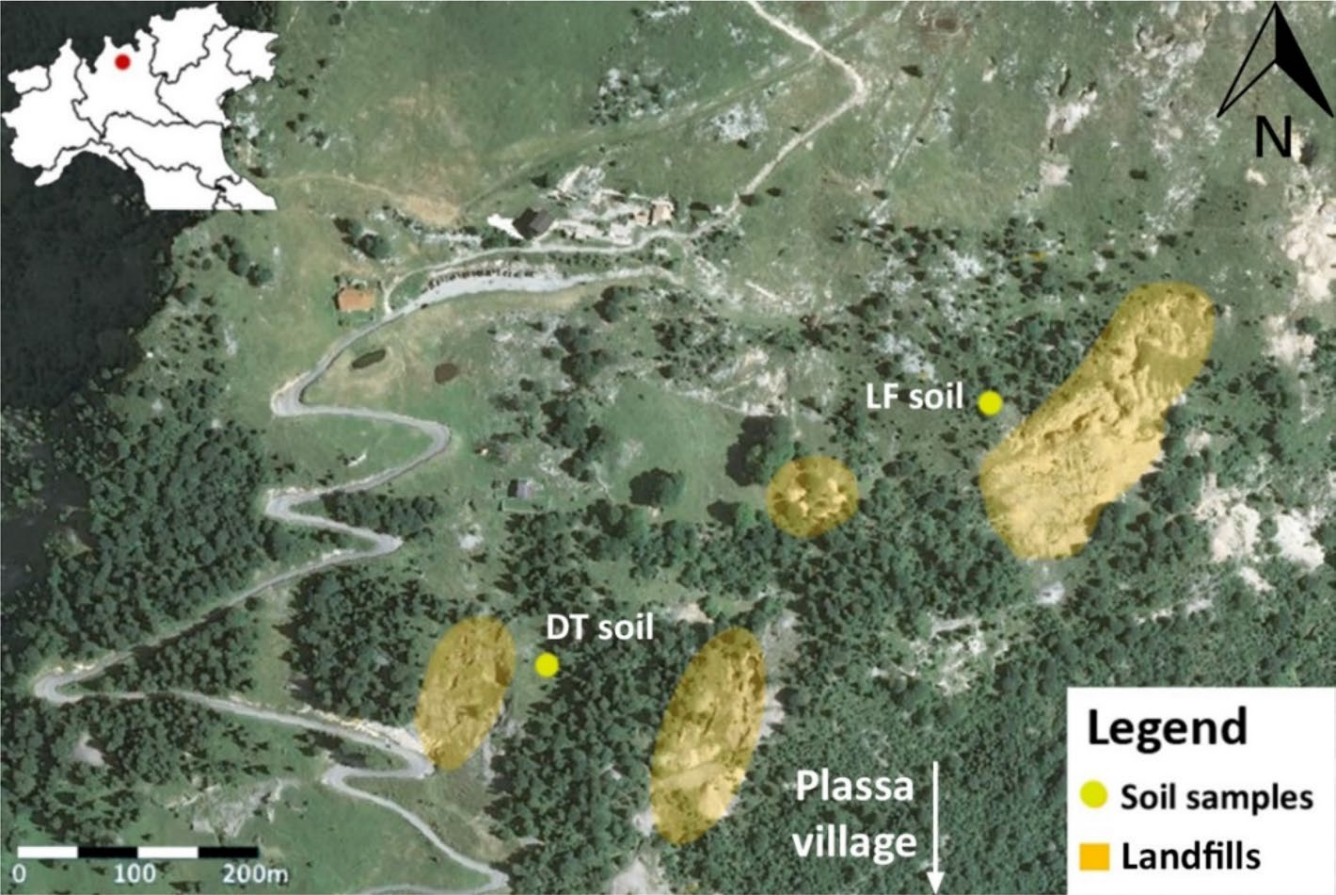
Geographic location of the sampling points (background image: Google, Digital Globe, 2018), adapted from Padoan et al. (2020)

Mine-affected soil samples were collected in the mining basin of Gorno, near the village of Plassa (PL) (45°54′55.1" N; 9°47′50.0" E), approximately 25 km northeast of the city of Bergamo in the Orobian Alps (Fig. 1) (Lombardia Region, Italy). The Gorno basin lies in the prealpine area, with strong daytime thermal excursions but limited annual thermal excursions and abundant precipitation (e.g., the average annual precipitation in the years 2016–2018 years was 1988 mm) (ARPA Lombardia 2020). The average air temperature is around 8 °C, and the climate is classified as temperate oceanic (Cfb), according to the Köppen–Geiger climate classification.

The mining district was known since the Roman Age, but the industrial exploitation of the site started during the nineteenth century and continued until 1982, encompassing different techniques and mining companies. Its primary mineralization includes sphalerite (ZnS) and galena (PbS), with minor occurrence of pyrite (FeS_2_), marcasite (FeS_2_), chalcopyrite (CuFeS_2_), and argentite (Ag_2_S) (Dino et al. 2018). As the secondary mineralization, composed of oxidation products of sphalerite, was preferred for ore exploitation, the rocks containing sphalerite and galena were separated and placed outside the tunnels, forming waste rock dumps spread across the whole district (Dino et al. 2018; Mehta et al. 2020). The waste rock dumps have not been remediated after the conclusion of the mining activities. The mining debris were then scattered across the surrounding areas during and after the very long period of exploitation and now most of the slope appears to be heavily contaminated, particularly around the former dumping areas, with Cd concentrations in the topsoil between 13 and 493 mg/kg and Zn values in the range 9–132 g/kg (Padoan et al. 2020). After the dismissal of the mining operation, the entire area was open to tourism and agriculture and pastures.

Following previous characterization studies (Padoan et al. 2020; Mehta et al. 2020), we selected and sampled two soil samples representative of the slope. The soils also were selected in view of their similarity in organic carbon and Fe content and their difference in pH and presence of carbonates.

The first soil was sampled near the biggest exposed landfill (LF) on the slope, in a pasture above the area with the exposed rock debris (Fig. 1). The second soil was sampled among the exits of old ducts and tunnels (DT), around which mining debris have been abandoned.

Soil samples were taken at 0- to 15-cm depth, by combining five subsamples for each one taken at the vertices and at the centre of a 2- × 2-m square and brought to the laboratory in plastic bags. Samples were air-dried, gently crushed, and sieved to < 2-mm diameter for the physicochemical characterization and to < 5 mm for column studies.

### Soils Characterization

The soil physicochemical features were determined as follows: the pH was determined potentiometrically in a 1:2.5 soil:water suspension; total carbon (TC) and nitrogen (TN) were determined by dry combustion with an elemental analyzer (NA2100, CE Instruments, Italy) and carbonates by volumetric method. Particle size distribution was determined via the sieve-pipette method. All analyses were performed according to the official Italian methods for soils (Colombo and Miano 2015).

Pseudo-total elemental contents were determined according to Italian legislation; on the samples ground to < 150 µm before analysis after *aqua regia* extraction (HCl/HNO_3_, 3:1 v/v) with microwave digestion of 0.2 g of soil (Milestone Ethos D, Italy) (Colombo and Miano 2015; Padoan et al. 2017). Concentrations of Fe, Mn, Zn, Cd, Ni, Cr, Cu, and Pb were determined by FAAS (PerkinElmer AAnalyst 400, Massachusetts). All analyses were performed in triplicate and accuracy was validated using Certified Reference Materials for *aqua regia*-soluble contents (CRM 141R and CRM 142R, Community Bureau of Reference, Geel, Belgium). CRM recoveries were always within 90–105% of the certified values. All reagents were of analytical grade.

Iron oxides forms were estimated using the dithionite-citrate-bicarbonate (Fe_DCB_) and the acid ammonium oxalate (Fe_Ox_) extracting solutions (Colombo and Miano 2015). Iron was determined in both extracts by FAAS. Metals extractable by CaCl_2_ were determined by FAAS in the extract obtained by shaking 5 g of sample for 2 h in 0.01 M CaCl_2_, using a 1:10 soil to solution ratio (Houba et al. 2000).

### Column Experiments

**Fig. 2:**
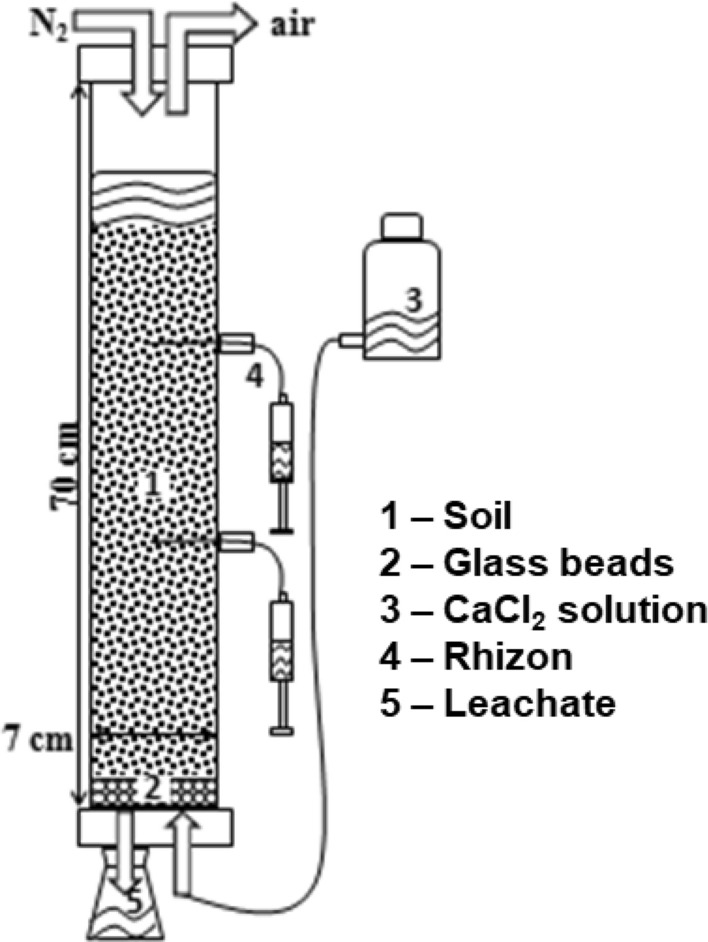
Experimental setup of the column mesocosms

To simulate flooding conditions, approximately 1.3 kg of each soil (sieved at 5 mm to limit clogging) was placed inside duplicate poly(methyl-methacrylate) columns as in Fig. 2 (70 cm in height and 7 cm internal diameter) (Balint et al. 2014).

Soils were waterlogged by using a solution containing 10 mM of CaCl_2_, 3 mM of lactose, and 10 mL of bacterial inoculum (Balint et al. 2014). Bacterial inoculum was extracted from paddy soil with a Ringer’s solution, and it was used to accelerate reduction onset after preliminary experiments in anaerobic chamber demonstrating its effectiveness. The solution was slowly added from the bottom of the columns to minimize preferential pathways. Once the soil had become saturated, it was flooded to approximately 10 cm above the surface. The columns headspace was then flushed with N_2_ to remove all the air and the columns were hermetically sealed. Two *Rhizon* samplers (Rhizosphere Research Products, The Netherlands) were inserted in each column at different heights. The first one collected pore water approximately at the centre of soil column, at 20-cm depth. The second was used to collect the supernatant water above the soil surface, at 5-cm depth.

The soil solution and the supernatant water were sampled at defined intervals up to 9 weeks after flooding. At the end of the experiment, the bulk soil solution was collected by draining each column. All collected solutions were immediately filtered at 0.45 µm, and an aliquot was used to spectrophotometrically determine Fe (II) using the 1,10-phenanthroline method (Loeppert and Inskeep 1996). Another aliquot was acidified at pH 2 using hydrochloric acid and stored in plastic tubes at − 20 °C for further analyses. Solutions were analyzed for total (Fe, Mn, Zn, and Cd) metal contents by FAAS and dissolved organic carbon (DOC) using Pt-catalyzed, high-temperature combustion (850 °C) after removing inorganic C (Elementar VarioTOC, Germany). Because measuring the Eh and pH would have implied serious disturbance to the column system, these two parameters were potentiometrically measured in a parallel small pot experiment where the column conditions were replicated.

A leaching test also was conducted to observe the Zn and Cd leaching potential in aerobic conditions—for example, after rain events to be compared to the anaerobic release data. To this aim, the columns were filled with approximately 1 kg of soil and brought to field capacity with a CaCl_2_ 10 mM solution, added as before, until a liquid film formed on the surface of the soil.

Columns were left open to air for 24 h and then drained before the onset of reductive conditions. The leachate was collected, filtered, acidified, and stored as described above for further analysis. The leachate was analysed for metals (Fe, Mn, Zn, and Cd) and DOC. In total, 15 leaching events were performed.

### Chemical Fractionation of Metals

Chemical fractions of Fe, Mn, Zn, and Cd in the two soils were determined using the modified BCR sequential extraction procedure described by Rauret et al. (2000) before and at the end of the column experiment in duplicate. The extracted fractions were operationally divided in:

- F1: exchangeable fraction, water, and acid-soluble metals (e.g., co-precipitated with carbonates) (0.11 M acetic acid).
- F2: reducible fraction, fraction of metals bound to Fe–Mn oxides (0.5 M hydroxylammonium chloride, at pH 1.5).
- F3: oxidisable fraction, bound to organic matter and sulfides (hydrogen peroxide, at 85 °C, then 1 M ammonium acetate at pH 2).
- F4: residual fraction, digested with *aqua regia*.

All suspensions were centrifuged, filtered, and analysed for Fe, Mn, Zn, and Cd by FAAS. Before and after each extraction step, samples were weighed to avoid an underestimation of the metal contents.

### Geochemical Modelling

To describe Zn and Cd partitioning between solid surfaces and liquid phases in the studied soils, we performed geochemical calculations using a multisurface modelling approach with the ORCHESTRA (Objects Representing CHEmical Speciation and TRAnsport) software (Meeussen 2003). This software includes three models for ion adsorption on reactive surfaces—i.e., the NICA-Donnan model for particulate (SOM) and dissolved (DOM) organic matter, the double layer model for short-range ordered Fe/Al(hydr)oxides, and a Donnan model for clay (Dijkstra et al. 2004). The generic setup of the model was similar to Pan et al. (2016), whereas we used the assumptions reported in Bonten et al. (2008) to model the reactivity of the adsorption surfaces. The solution speciation was calculated after 3 days of submersion, after 20 days, and at the end of the experiment after 60 days.

Data input for the model were: (1) the measured Eh and pH values; (2) the available concentrations of the competing ions Ca^2+^, Mg^2+^, NO_3_^−^, NO_2_^−^, SO_4_^2−^, PO_4_^3−^, measured after 0.01 M CaCl_2_ extraction with ion chromatography (Dionex DX-500, California); (3) the Zn, Cd, and DOC concentrations as measured in the soil solution, (4) the reactive Fe and Mn (hydr)oxide, determined using the ammonium oxalate extraction, (5) the total concentrations of Zn and Cd and those of Ni, Cr, Cu, and Pb to represent a total reactive pool of trace metals that could react with OM, ions, and oxides. To refine the model, we chose to include in the calculations all the minerals controlling the solubility of Fe, Mn, Ca, Pb, Cu, Cd, and Zn, including sulfides, carbonates, and (hydr)oxides.

## Results and Discussion

### Soil Properties

The main physicochemical properties and heavy metal pseudo-total contents of the soils used in this study are presented in Table 2. As explained above, the soils were specifically chosen to have two samples representative of the similarities and the differences present along the slope.

**Table 2:** Physicochemical properties and pseudo-total heavy metal concentrations in soils

	Unit	LF soil	DT soil	Legislative limit^a^
pH_H2O_		7.5	6.9	
Carbonates	%	32	7.4	
C_tot_		10.5	9.1	
C_org_		4.2	7.7	
N_tot_		0.5	0.7	
Clay		2	3	
Silt		13	12	
Sand		85	85	
Fe	g kg^−1^	34	33	
Fe_DCB_		26	30	
Fe_Ox_		7.5	9.9	
Mn		5.2	3.2	
Zn		112	65	0.15
Cd		0.36	0.16	0.002
Cu		0.22	0.11	0.12
Cr		0.03	0.04	0.15
Pb		0.16	0.49	0.10

^a^According to the law (MATTM 2006), for green and residential areas

Both soils were categorized as loamy sand, according to USDA texture classification, with neutral to slightly alkaline pH. These pH values are due to the carbonates, which are present in both soils in considerable amount. The LF soil had the highest value and a corresponding pH higher than DT.

Organic carbon (C_org_) content was calculated from the difference between total carbon and carbonates. The C_org_ of both soils was high as is typically observed in many mountain soils, particularly in grassland and natural treeline ecosystems (Leifeld et al. 2009; Hagedorn et al. 2010).

Heavy metal concentrations in the soils were very high in consideration of the sampling location (Dino et al. 2018) with Zn, Cd, Cu, and Pb higher than the limits established by Italian legislation (MATTM, 2006) for green and residential areas, with Zn and Cd also exceeding, by far, the limits for industrial areas (1.5 g kg^−1^ for Zn and 0.015 g kg^−1^ for Cd).

Iron and manganese, the key elements for the soil response to reduction, appeared as in the range of the area, although Mn concentrations were high compared with other areas (Alloway 2013).

Free iron oxides (Fe_DCB_) content also was within the range for the soils of this climatic zone, whereas Fe_Ox_ was the 29% and 33% of Fe_DCB_, indicating a moderate degree of soil evolution (Arduino et al. 1986). After the initial characterization, this study focused on Fe, Mn, Zn, and Cd as Cu, Cr, and Pb posed a considerably lower environmental risk.

### Leaching Experiments

Results for Cd and Zn concentration in the bulk water during the leaching trials are displayed in Table 3. The release of metals from the soils under aerobic conditions was remarkably high on every leaching event. The DT soil released higher amounts of mobile metals, particularly Cd, whose solution concentrations were twice those of LF in almost every events.

**Table 3:** Zinc and Cd leachate concentrations (mg l^−1^) during aerobic leaching trials

Event	Cd		Zn	
	LF	DT	LF	DT
1	0.16	0.18	9.3	20.3
2	0.18	0.22	11.5	22.2
3	0.13	0.15	15.2	15.1
4	0.13	0.17	12.2	14.2
5	0.12	0.21	11.9	11.4
6	0.12	0.25	11.4	9.8
7	0.12	0.29	12.4	10.5
8	0.11	0.28	11.2	9.7
9	0.10	0.19	12.4	9.8
10	0.10	0.20	11.7	9.6
11	0.15	0.29	16.4	18.6
12	0.17	0.37	18.3	27.1
13	0.17	0.31	17.7	21.9
14	0.16	0.18	12.0	15.4
15	0.20	0.23	13.4	18.3

Leaching solution consisted in 10 mM CaCl_2_

No trend (Table 3) was observed for the selected elements, with Cd and Zn amounts fluctuating between the trials, pointing to a release due to the prompt dissolution of the more mobile salts, favoured from the very high concentrations of metals present in the soils.

Iron and Mn concentrations also were assessed to have information on the possible onset of the anaerobiosis during the trials, as an increasing release would have been a symptom of reductive conditions. A decreasing trend was noted for Fe concentrations (reported in the Electronic Supplementary Information [ESI]), reduced under the detection limit (0.01 mg/l) after 7 cycles in both soils. Manganese concentrations seemed to increase in both soils after each cycle of wetting and drying; this behaviour is coherent with the increase of a leachable fraction due to the dissolution–precipitation phenomena.

The average concentrations of 0.14 mg l^−1^ of Cd leached from LF and 0.23 mg l^−1^ from DT are two order of magnitude higher than the maximum concentrations allowed in groundwater by the national legislation (MATTM 2009) (0.005 mg l^−1^ for Cd and 3 mg l^−1^ for Zn). Thus, the carbonatic component of the soil, which should decrease the solubility of the metals, seems not to protect the environment from the rapid release of potentially toxic elements under anaerobiosis.

The total amounts of metals released during the experiment were 0.29 mg kg^−1^ of Cd and 27 mg kg^−1^ of Zn leached from LF, whereas 0.63 and 42 mg kg^−1^ were released from DT soil. Using these quantities, we calculated a theorical maximum of Zn and Cd potentially leached from the top 15 cm of 1 m^2^ of soil under an amount of rain equivalent to 1 year (using a bulk density of 1.3 g cm^−3^ and the average values between the two soils for the metals), obtaining 63 g of Cd and 4,700 g of Zn moving each year from the top to subsoil and, eventually, ending up in the ground water. The experimental design does not allow to generalize due to very different factors acting in a natural site, such as the presence of a carbonate layer or the morphology of the site. As an example, the actual risk could be lower as the sampling site is a steep slope, so the infiltration rate of the rain could be low. Still, in that case the erosion could increase the risk of transport of metals via runoff, and the particles could end up in an anoxic site, such as a water body.

### Flooding Experiment

**Fig. 3:**
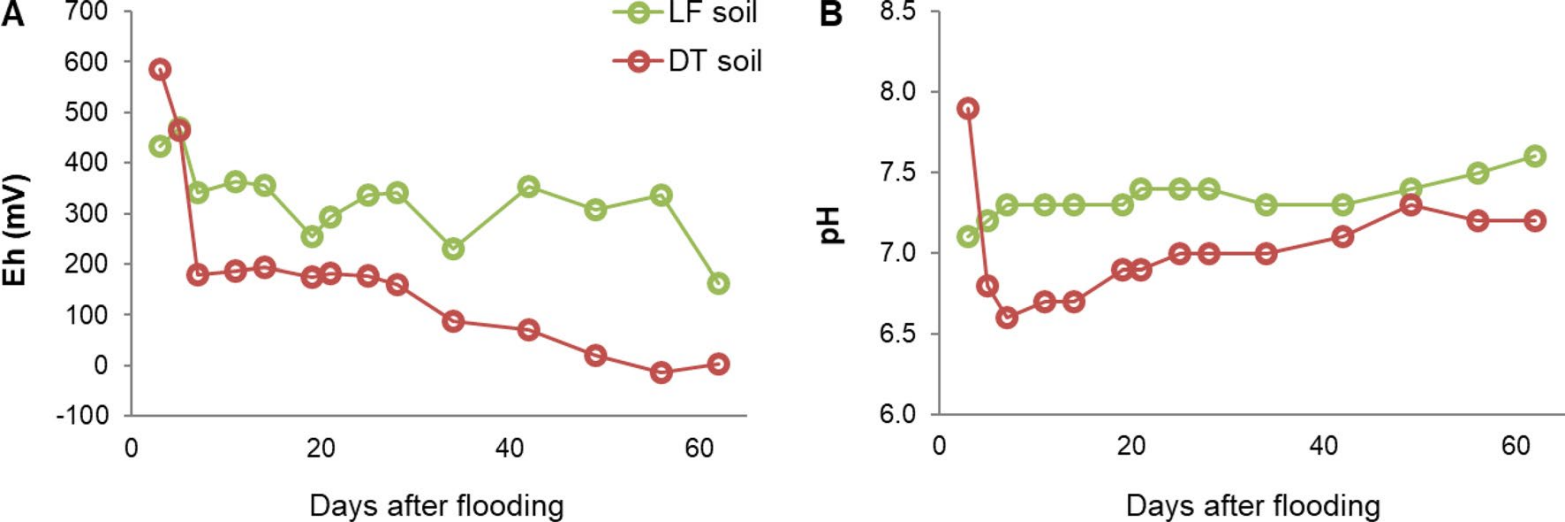
Eh and pH values of the LF and DT soil solutions during the 62 days of the flooding experiment

The values of redox potential (Eh) and pH obtained over the two months (62 days) of experimental flooding are presented in Fig. 3.

The Eh decreased rapidly in both soils during the first days of the experiment (Fig. 3a), following a behaviour common to flooded soils (Ajmone-Marsan et al. 2019). LF maintained higher Eh values over time, indicating lower reduction intensity in the system, whereas DT showed Eh values characteristic of a reduced environment already during the first week. The pH (Fig. 3b) increased in both soils since the reduction process leads to the consumption of H^+^ ions.

The waterlogging resulted in a DOC increase in both soils during the first days on flooding, together with the decrease of Eh. The values become almost constant after the second week of reduction; the average is 391 ± 39 mg l^−1^ in LF soil and 561 ± 45 mgl^−1^ in DT soil. This behaviour was already observed in similar situations and is probably due to both the dissolution of mobile hydrophilic DOC compounds during the first days and to the release of the organic matter (OM) bound to the oxyhydroxides surfaces (Shaheen et al. 2014; Pan et al. 2014). Iron and Mn minerals strongly adsorb OM, but once the soil is water-saturated, the increase in pH and the anoxia diminish the positive surface charge of the oxyhydroxides and force the microorganisms to switch and use Mn and Fe as electron acceptors, causing the release of the associated OM.

**Fig. 4:**
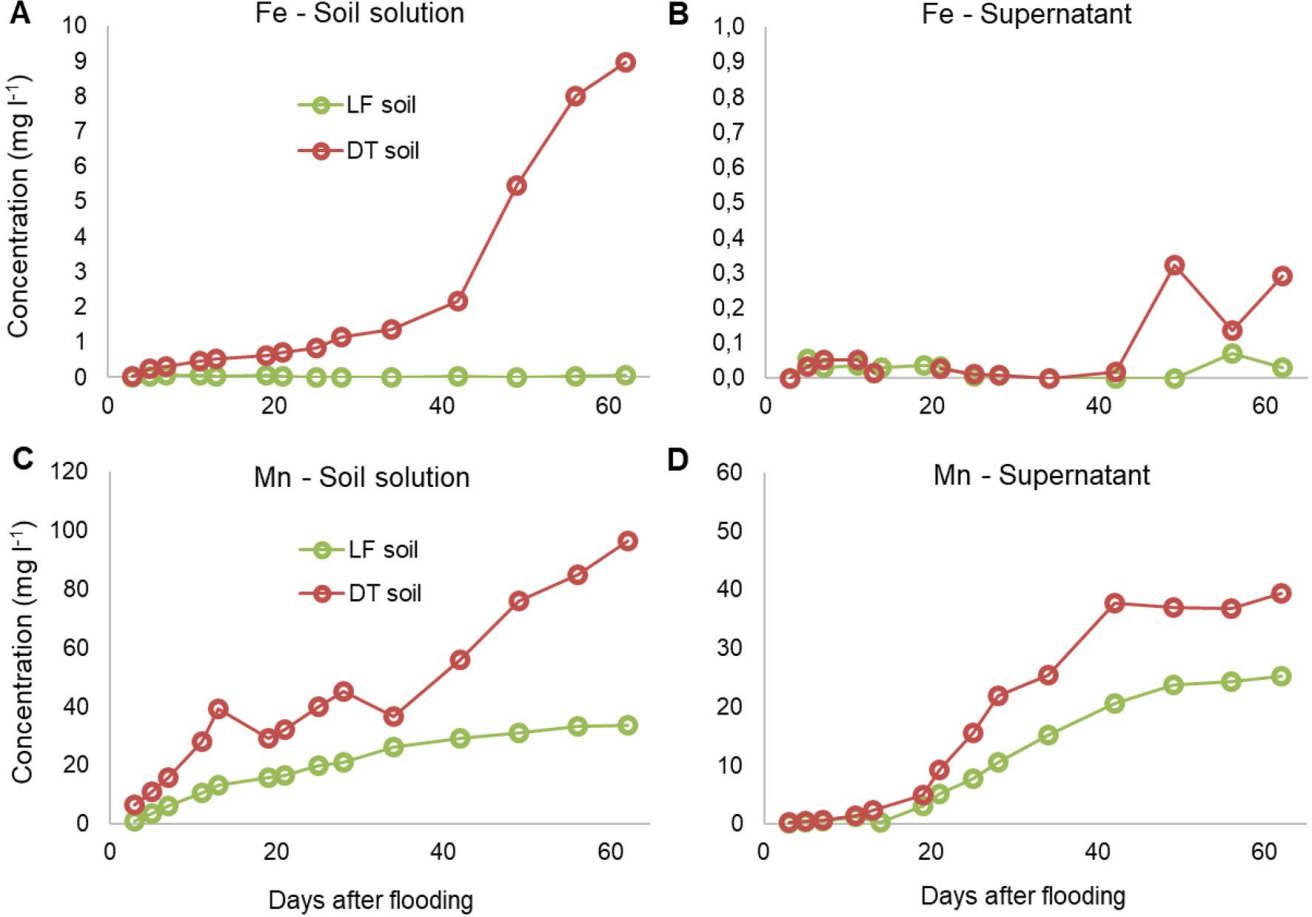
Average contents of Fe (**a**, **b**) and Mn (**c**, **d**) in the soil solution and in the supernatant solution over 62 days of flooding (note that the *Y* axis scale for the supernatant solution is a fraction of that of the soil solution)

Concentrations of Mn and Fe were higher in DT than in LF in both soil solution, measured at the centre of the soil columns, and in supernatant solutions (Fig. 4). In the DT soil, Mn reduction started immediately, with a strong increase in the soil solution observed after 5 days of submersion; Fe^3+^ was slowly reduced until the 5^th^ week of experiment when soil solution concentrations started to increase. In the LF soil, Mn levels in the soil solution increased gradually during all the experiment, whereas the Fe^3+^ reduction did not start during the entire study period.

This behaviour was likely due to the high Mn contents of the soils; since Mn oxides reduction precedes ferric oxides, they would buffer the redox reaction, stabilizing the Eh at higher values and delaying the Fe release in soil solutions (Ponnamperuma 1972). This tendency was more evident in LF soil, containing more Mn oxides than DT soil.

In both soils, and particularly in LF, the low presence of Fe^2+^ and Mn^2+^ in solution could be due to the precipitation of carbonates. Concentrations in the supernatant solution followed the described trends, with a delay due to the slower soil to water diffusion at the interface.

**Fig. 5:**
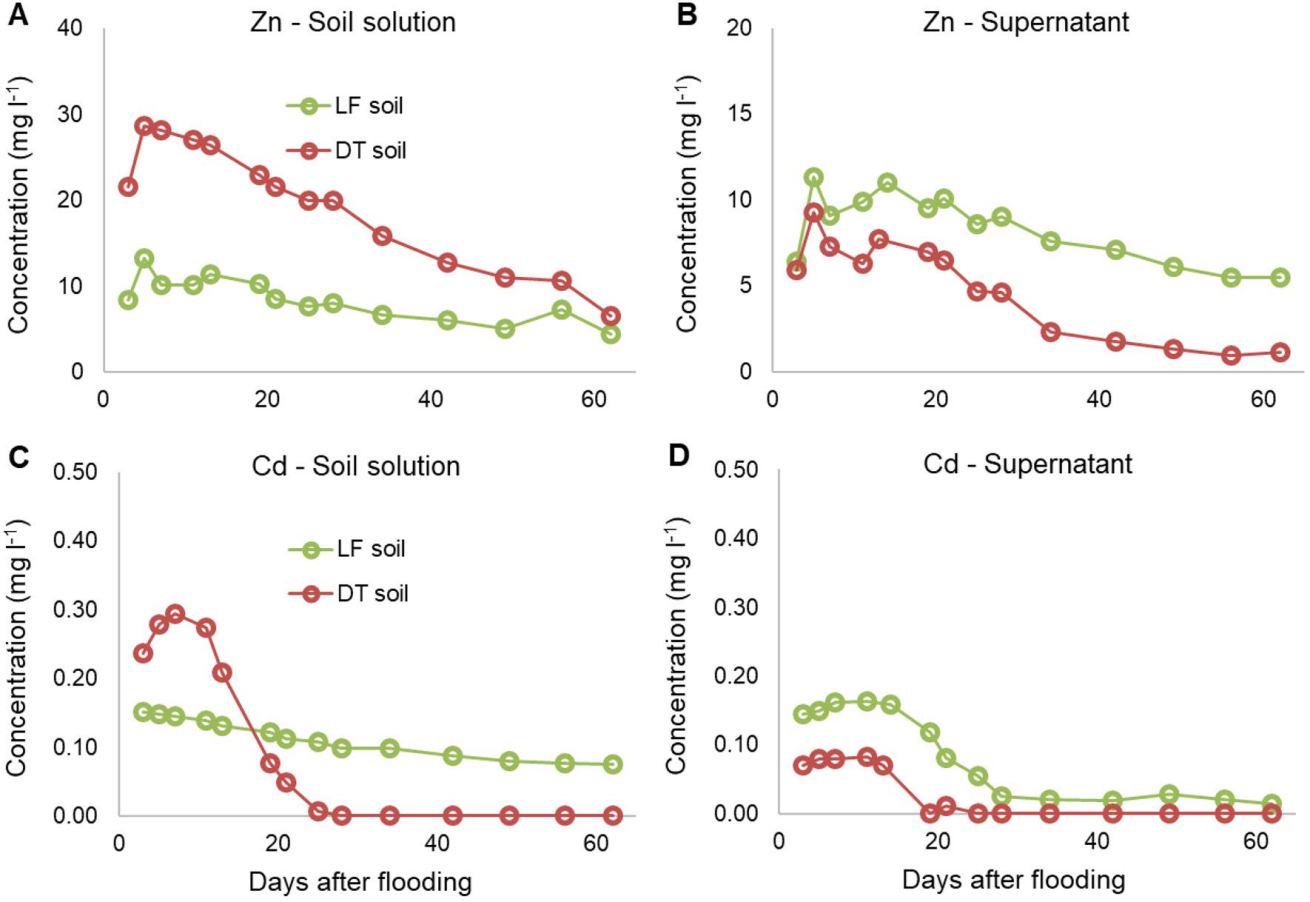
Average contents of Zn and Cd in **a**, **c** soil solution and **b**, **d** supernatant solution in soils over the study period

Contrary to the slow release of Mn and Fe, Zinc and Cd concentrations in soil solutions (at the centre of the soil column) increased to peak during the first week of flooding in both soils (Fig. 5). Released concentrations during the first days were similar to ones released during the aerobic leaching trials reported above, with peak concentrations slightly higher, pointing to a rapid solubilization of the more mobile forms of Zn and Cd. Concentrations started to decrease from the second week until the end of the experiment, almost linearly for Zn in both soils and for Cd in LF, whereas in DT the Cd concentration rapidly decreased until zero after 28 days.

The decrease of solution concentrations could be due to different factors, such as the high concentration of natural OM, carbonates, and sulphates deriving from the formerly extracted minerals. OM could form surface complexes with Zn and Cd, decreasing the solubility of these ions. Cadmium, in particular, can form strong inner-sphere complexes with OM and under reduction Cd speciation could be dominated by humic acid complexes (Karna et al. 2016). This could be the case of LF soil, where both Zn and Cd were negatively correlated with the DOC (*r* = − 0.78 for Zn and *r* = − 0.85 for Cd).

Other reasons for the decrease of soil solution concentrations in both soils could have been the precipitation of Zn as Zn(OH)_2_ due to pH increase, or the precipitation of ZnCO_3_. In highly contaminated calcareous soils, in fact, the most important forms of Zn minerals have been found to be Zn-layered double hydroxides and Zn carbonates, amorphous or as hydrozincite (Jacquat et al. 2008; Khaokaew et al. 2012), or the metal could have been co-precipitated with Fe and Mn ions released after oxides reduction.

A high soil carbonate content also could affect the adsorption of the elements, as in alkaline soils Zn is adsorbed more intensely by soil components (Lindsay 1972) due to the specific adsorption on the surface functional groups that have pH-dependent charge (Frohne et al. 2011; Alloway 2013).

It is interesting to note that LF had twice the total Zn than DT (Table 2) but under anaerobiosis the situation was reversed, with the amount of Zn released to the soil solution from DT becoming greater than LF. This could be due to the different binding forms of the Zn the soils and to the carbonates content—almost five times higher in LF than in DT—associated to the high initial concentrations of Zn.

Cadmium concentrations in soil solution displayed different tendencies in the two soils. In LF, the concentration decreased gradually over time, whereas in DT a rapid decrease until zero was observed (Fig. 5c). An acceleration of the precipitation of Cd phases in this soil would be in line with its faster onset of anaerobic conditions. Khaokaew et al. (2011) demonstrated that, in alkaline flooded soils from Thailand, species controlling Cd solubility were carbonates (Cd–CaCO_3_ and CdCO_3_), with only a small contribution from CdS, whereas in the case of contaminated mine waste material under prolonged reduction, Karna et al. (2016) found the formation of Cd-sulphates and Cd-sulphides in the samples.

Parallel to the soil solution, the supernatant solution was sampled during all the experiment.

(Figs. 5b and 5d). In LF soil, the concentrations of metals followed the same trend observed in the soil solution, with an initial increase followed with a steady decrease. Conversely, in DT the diffusive flux was less intense and the concentrations in the supernatant were lower than ones in LF soil; but even at lower concentrations, a concordance between supernatant and soil solution curves slopes was observed. The diffusion from the soil lead to Cd and Zn concentrations in the supernatant solution higher than the groundwater legislative limit for the first 3 weeks of flooding, with Cd being 10 to 30 times higher than the threshold.

These data are extremely important from an environmental point of view, because during an occasional flooding the metals could be released promptly to the soil solution, but also to the aquifer in contact with the soil due to the solute diffusion from the soil to the water layer.

#### BCR Sequential Extraction

**Fig. 6:**
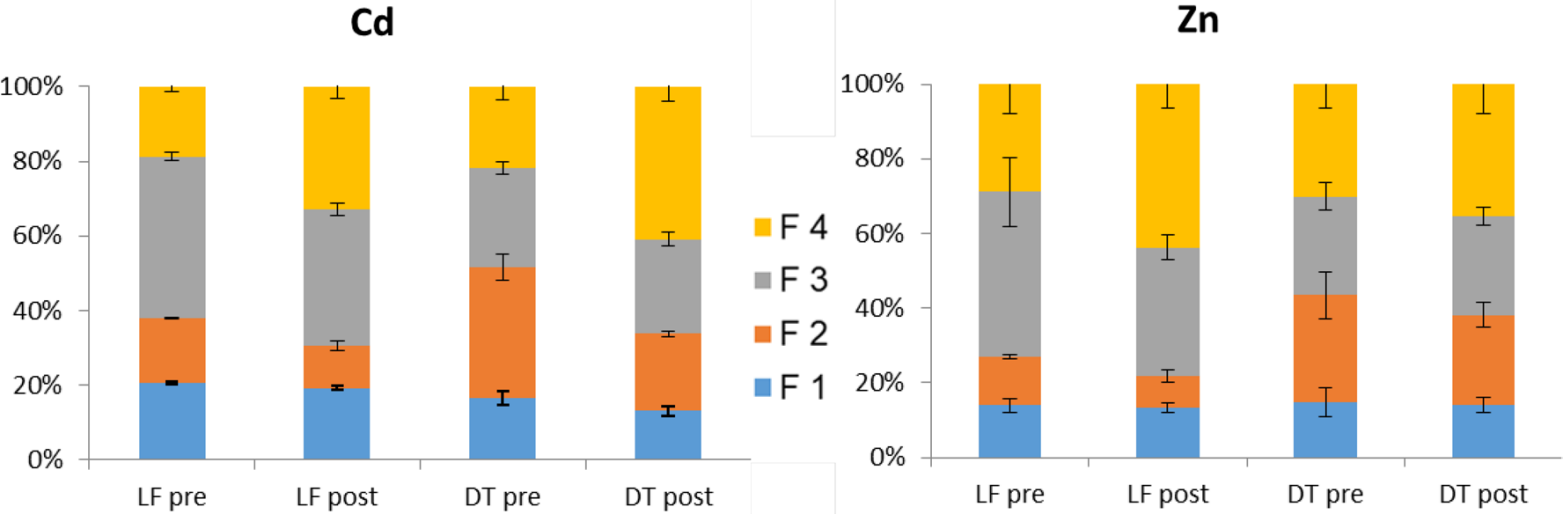
Distribution of Zn and Cd among the exchangeable fraction (F1), bound to Fe and Mn oxyhydroxides (F2), bound to organic matter and sulphides (F3) and residual fraction (F4) obtained by BCR sequential extraction of the LF and DT soils before (pre) and after (post) the flooding, expressed as percentage of the pseudo-total amount

The sequential BCR protocol was applied before and after the flooding experiment to have additional information on the effect of the reductive conditions on the lability of the metals (Fig. 6). Results for Mn and Fe are reported in the ESI.

Analysing the average distribution of the metals—based on the proportion of each fraction in the total concentration—in both soils Zn and Cd were predominantly present in the potentially mobilizable fractions (F1 + F2 + F3). The metals responded in a similar way to waterlogging. The percentage of elements extractable in the fraction F1 (exchangeable and acid-extractable) was comprised between 10–20% and did not changed after the flooding for Cd, Zn, and Fe.

The highest percentages of Zn and Cd in DT soil were extracted in the F2 fraction, whereas in LF soil, Zn and Cd were preferentially retained in the F3 (organic), which means a tendency to remain in less available forms.

These results were consistent with the amounts of metals released in solution during the anaerobic trial. DT soil, where the metals were adsorbed mostly on oxides, released twice the Zn and Cd amount mobilized from LF soil, probably because of the oxides dissolution. In LF, the inner-sphere complexation with the organic matter would strongly limit the solubility and the mobility of Zn and Cd due to the strength and irreversibility tendency of the binding.

After the flooding, both fractions varied slightly, whereas only the residual fraction (F4) increased, displaying a shift in the availability of the metals due to the extended reduction. This fraction summed for Zn and Cd from 33 to 44% of the total concentrations, increasing proportionally to the expected decrease of the forms associated to Fe and Mn (F2).

In both soils, Mn was predominantly extracted in the reducible (F2) fraction (Fig. 1SM), as it normally occurs in soils with a pH higher than 6. During the wet-dry cycle, released Mn^2+^ precipitated probably as both amorphous oxides (Ajmone-Marsan et al. 2019) and carbonates, as after the reduction the fraction F1 (exchangeable and acid-extractable) markedly increased in both soils while F2 decreased. Conversely, Fe had > 90% of the element extractable only through *aqua regia* extraction (F4) before the reduction, whereas after the reoxidation, in both soils F2 doubled, as newly precipitated Fe will form poorly ordered Fe-hydroxide minerals (Balint et al. 2014; Ajmone-Marsan et al. 2019). This was confirmed by the decrease in the ratio between the Fe_DCB_ and the Fe_ox_, normally attributed to the increase in the amorphous fraction of iron oxides (Arduino et al. 1986).

#### Geochemical Modelling

To further investigate the relative importance of the different reactive surfaces and the solubility dynamics of Zn and Cd in the soil solution as a function of time, we modelled the solution speciation and the precipitation dynamics during the flooding experiment (after 3, 20, and 60 days of submersion).

In the soil under anaerobic conditions, both metals were calculated to be predominantly present as minerals during all the trial, with the large majority (higher than the 90%, not reported) of the soluble Zn and Cd predicted to precipitate in both soils as carbonates after 3 and 20 days.

Sixty days after the onset of the anaerobiosis, no sulphide precipitation was predicted in LF soil; the decrease to almost zero of the adsorbed Zn and Cd fractions was calculated as due to the precipitation of carbonates. Conversely, in DT, where a much lower redox potential was detected, metals were predicted to precipitate as Zn and Cd carbonates during the first days, whereas a sulphide precipitation was calculated after 60 days of submersion, using as available sulphur the SO_4_^2−^ in the soil solution, although low (23 and 58 µmol L^−1^ in LF and in DT soil, respectively) and insufficient to react with all the soluble metals.

**Fig. 7:**
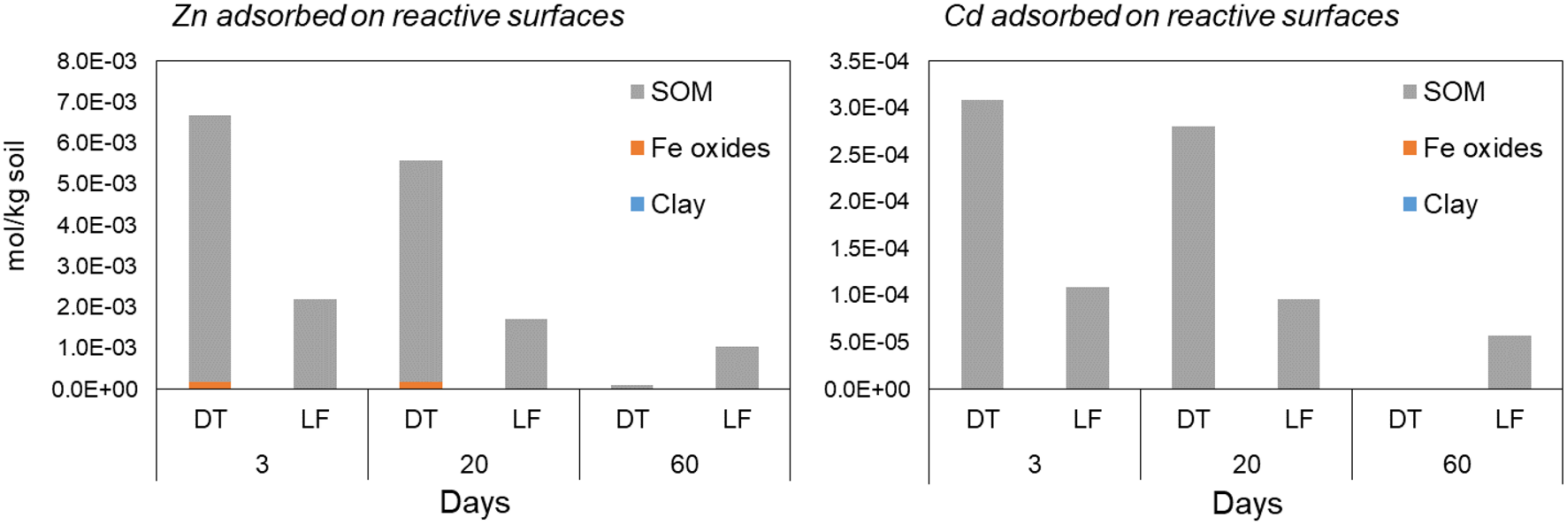
Zinc and Cd speciation in the solid phases of DT and LF soils after 3, 20, and 60 days of flooding (mineral precipitation not reported)

The calculated Zn and Cd speciation in solution and sorption on the major types of soil reactive surfaces—soil organic matter (SOM), iron and manganese (hydr)oxides, and clays—are reported in Fig. 7.

Among adsorption surfaces, SOM is the dominant surface in the binding of Cd and Zn, and the only component predicted to have an influence on both elements, probably because of the very large quantity of OM present in these mountain soils. This behaviour also was predicted and observed for aerobic and anaerobic conditions in previous studies (Degryse et al. 2011; Pan et al. 2016; Ren et al. 2017).

Adsorption to clays and Fe oxides seem to have small influence on the concentrations, playing a minor role for Zn and negligible for Cd in both soils. These results are in agreement with the observed release to the soil solution, where the metals were not related to the Fe or the Mn release.

**Fig. 8:**
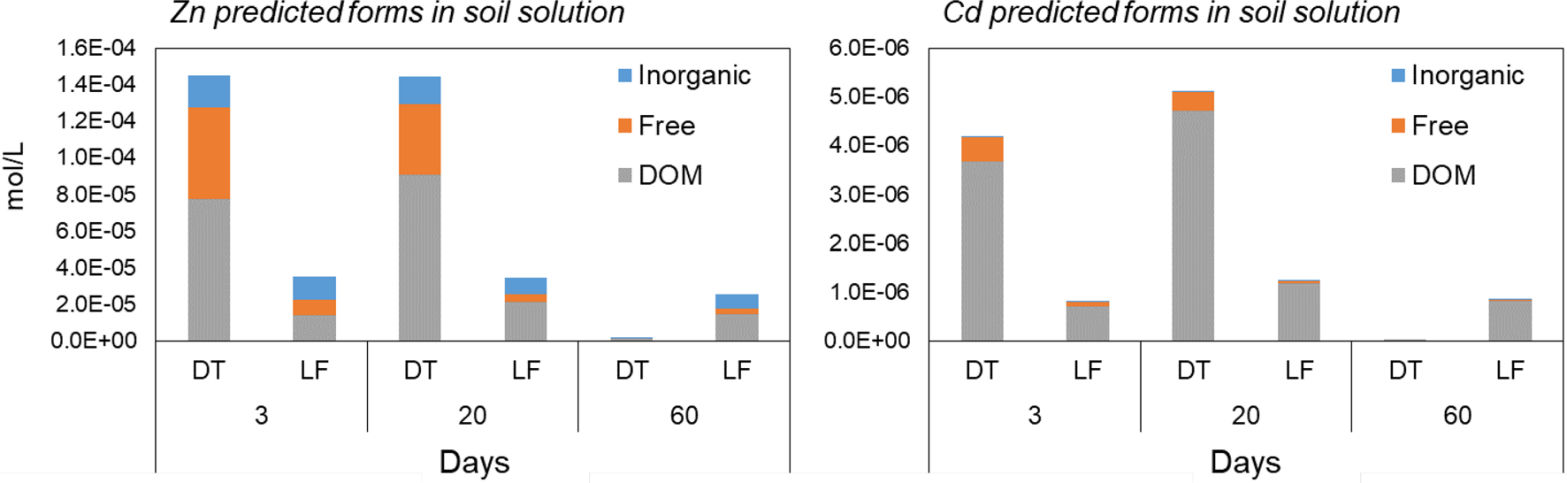
Zinc and Cd speciation in soil solution as predicted from the ORCHESTRA model

The Zn and Cd soil solution speciation was partitioned by the model into three classes: inorganic complexes, organic (DOM), and free metal ions (Fig. 8). Due to the prolonged anoxic conditions, we performed different calculations, including and excluding the precipitation of all the minerals controlling the solubility of Cd and Zn, comprising sulphides (Pan et al. 2016).

In both soils, at near neutral pH, Zn and Cd were calculated to be predominantly complexed by DOM, as found by Dijkstra et al. (2004). For Zn, inorganic complexes were predicted to be important, probably because of CO_3_ complexes, as their importance was much higher in LF soil, which had a high carbonate concentration. The presence of both elements as free ions in solution was calculated to be significant during the first days after the flooding, becoming dominant over inorganic complexation in the case of Cd.

**Fig. 9:**
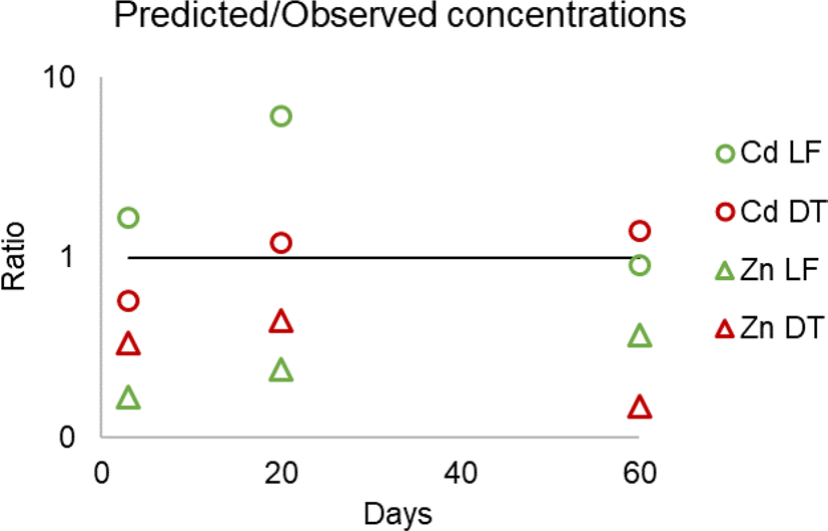
Ratio between predicted and observed soil solution concentrations of Cd and Zn. The line represents a one to one ratio, indicating no difference between modelled and analysed concentrations. A lower ratio represents an underestimation of the soil solution Zn or Cd concentrations

The simplest way to validate model predictions is to compare calculations with the observed metal concentrations in the soil solution. The model considering sulphide precipitation reasonably predicted the observed concentrations, as values were of the same order of magnitude of the observed ones (Fig. 9).

Particularly, Zn concentrations were underestimated in both soils because of a calculation of a slightly higher precipitation rate of sulphides, and the highest error was calculated for DT soil after 60 days, where the formation of (Zn,Fe)S (Otavite and Sphalerite) was predicted. Regarding Cd, predicted values were similar to observed ones for LF soil and good for DT, predicting the formation of CdS and the soil solution concentrations reaching almost zero in both soils after 60 days. The overestimation of the solubility in the LF sample after 20 days could have been due to the underestimation of Cd-carbonates precipitation or to specific metal retention processes on carbonated and (hydr)oxides not included in the generic adsorption models (Davis et al. 1987; Pan et al. 2016).

The calculated behaviour was consistent to one observed in the columns, giving interesting indications on the speciation not predicted from the sequential extraction schemes, such as the importance of the sulphide precipitation also at Eh values near zero. Thus, the use of multisurface modelling could be a valuable tool to have an indication of the speciation in solution also in the case of anoxic conditions, condition rarely assessed in previous studies.

## Conclusions

In this study, the potential release of Cd and Zn subsequent to a flooding in mine-contaminated soil of a mountainous area was evaluated using leaching and flooding experiments, sequential extractions and multisurface modelling. The studied soils, collected from grasslands near mining residues, were heavily contaminated but rich in carbonates and organic matter, two characteristics possibly preventing the transfer of the metals to the environment.

The leaching trials showed a high release of Zn and Cd to drainage waters from the soils in case of intense rains, with metals possibly ending up in the environment via groundwater or via surface runoff, due to the steep slopes. Thus, the carbonatic component of the soil seemed not to protect the environment from a rapid discharge of the metals on flooding.

During the continuous flooding experiment, metals were dissolved in solution during the first days and then started to precipitate, before as carbonates and then, in DT soil, also as sulphides, according to the modelling results. The release of Zn and Cd was found to be associated to carbonates and OM. In fact, the less polluted soil (DT) released twice the Zn and Cd amount mobilized from LF soil due, probably, to the lower amount of OM-associated metals as found from the sequential extraction. This was confirmed by the model results, where, besides precipitation, both adsorbed and soil solution metal speciation were calculated to be dominated from OM complexes. In view of the results, the use of multisurface modelling coupled with extractions and laboratory experiments could provide useful indications on the solution speciation in the case of flooding episodes, to estimate the metals release to the environment.

## Electronic supplementary material

Below is the link to the electronic supplementary material.

Supplementary material 1 (PDF 91 kb)
