# Supplementary material for: Potential Release of Zinc and Cadmium From Mine-Affected Soils Under Flooding, a Mesocosm Study
Source: Arch Environ Contam Toxicol. 2020 Nov 11;79(4):421–34. doi: 10.1007/s00244-020-00777-0 (PMC7688597; doi:10.1007/s00244-020-00777-0)
Supplement: Supplementary file 1 — Supplementary material 1 (PDF 91 kb) [file 244_2020_777_MOESM1_ESM.pdf]

Supplementary Information for:

Potential release of zinc and cadmium from mine-affected soils under flooding

Elio Padoan[1]*Aline Hernandez Kath[1,2], Ledemar Carlos Vahl[2], Franco Ajmone-Marsan[1]

[1] Dipartimento di Scienze Agrarie, Forestali e Alimentari, Università degli Studi di Torino, Grugliasco, Italy

[2] Soil and Water Management and Conservation Postgraduate, University Federal of Pelotas, Pelotas, Brazil

*Corresponding author:

Elio Padoan

Dipartimento di Scienze Agrarie, Forestali e Alimentari

Largo Paolo Braccini 2, 10095, GRUGLIASCO (Torino) – Italy

Ph: +39 011 670 8517

Email: elio.padoan@unito.it

ORCID ID: 000-0002-9211-2506

Table 1. Iron and Mn leachate concentrations (mgl-1) during the leaching experiments.

**Event**	**Fe**		**Mn**	
	LF	DT	LF	DT
**1**	0.06	0.10	0.14	1.8
**2**	0.04	0.08	0.36	3.6
**3**	0.10	0.05	3.2	2.1
**4**	0.03	0.09	4.7	4.6
**5**	0.02	0.03	4.5	7.8
**6**	0.04	0.06	5.7	10.0
**7**	0.00	0.00	8.5	9.6
**8**	0.00	0.00	10.6	12.4
**9**	0.00	0.00	10.9	9.5
**10**	0.00	0.00	12.6	12.4
**11**	0.00	0.00	10.6	15.7
**12**	0.00	0.02	13.0	24.4
**13**	0.00	0.01	12.6	25.5
**14**	0.00	0.01	5.4	14.3
**15**	0.03	0	6.2	17.0

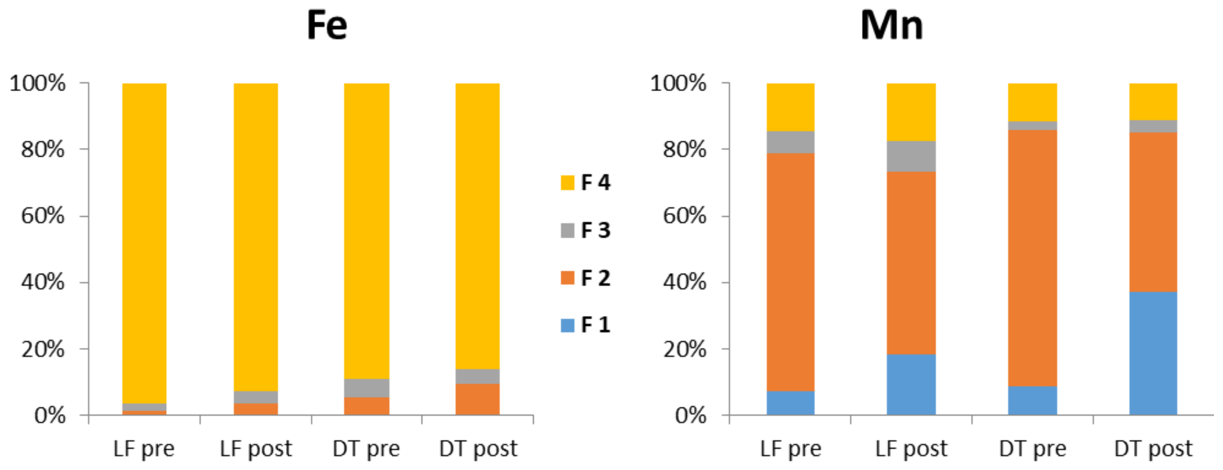

Figure 1. Distribution of Fe and Mn among the exchangeable fraction (F1), bound to Fe and Mn oxyhydroxides (F2), bound to organic matter and sulfides (F3) and residual fraction (F4) obtained by BCR sequential extraction of the LF and DT soils, expressed as percentage of the pseudo-total amount.
